# The role of single N-glycans in proteolytic processing and cell surface transport of the Lassa virus glycoprotein GP-C

**DOI:** 10.1186/1743-422X-3-41

**Published:** 2006-05-31

**Authors:** Robert Eichler, Oliver Lenz, Wolfgang Garten, Thomas Strecker

**Affiliations:** 1 Institut für Virologie der Philipps-Universität Marburg, Hans-Meerwein-Str. 3, 35037 Marburg, Germany; 2 Abbott GmbH & Co KG, Max-Planck-Ring 2, 65205 Wiesbaden, Germany; 3 Tibotec BVBA, Gen De Wittelaan L 11B 3, 2800 Mechelen, Belgium

## Abstract

Lassa virus glycoprotein is synthesised as a precursor (preGP-C) into the lumen of the endoplasmic reticulum. After cotranslational cleavage of the signal peptide, the immature GP-C is posttranslationally processed into the N-terminal subunit GP-1 and the C-terminal subunit GP-2 by the host cell subtilase SKI-1/S1P. The glycoprotein precursor contains eleven potential N-glycosylation sites. In this report, we investigated the effect of each N-glycan on proteolytic cleavage and cell surface transport by disrupting the consensus sequences of eleven potential N-glycan attachment sites individually. Five glycoprotein mutants with disrupted N-glycosylation sites were still proteolytically processed, whereas the remaining N-glycosylation sites are necessary for GP-C cleavage. Despite the lack of proteolytic processing, all cleavage-defective mutants were transported to the cell surface and remained completely endo H-sensitive. The findings indicate that N-glycans are needed for correct conformation of GP-C in order to be cleaved by SKI-1/S1P.

## Background

Lassa virus belongs to the family of *Arenaviridae *which includes other important human pathogens like Machupo virus, Junin virus, Guanarito virus and Sabia virus as well as the prototype of this family, Lymphocytic Choriomeningitis virus (LCMV). Lassa virus is endemic in certain parts of West Africa and causes an estimated 150 000 clinical cases of a systemic viral illness per year, with a mortality rate of 10–15% [1]. Lassa fever is not only a public health concern in endemic areas, it is also sporadically exported to other parts of the world [2].

Lassa virus is an enveloped virus with a bi-segmented RNA genome which encodes four viral proteins in an ambisense coding strategy: the glycoprotein precursor preGP-C, a nucleoprotein (NP), the viral polymerase L and the matrix protein Z (for review see [3]). The Lassa virus glycoprotein is translated as an inactive precursor (pre-GP-C) into the lumen of the endoplasmic reticulum and is then cotranslationally cleaved into GP-C and a stable signal peptide [4]. The latter one plays an important role in the subsequent posttranslational maturation cleavage of GP-C into its subunits GP1 and GP2 [5,6]. Lassa virus GP-C is cleaved at the C-terminus of a non-basic amino acid residue of the cleavage motif R-R-L-L by the subtilase SKI-1/S1P, which is unusual for fusiogenic glycoproteins of enveloped viruses [7]. So far, only viral glycoproteins of the arenavirus family and the glycoprotein of the Crimean-Congo hemorrhagic fever virus belonging to the family of *Bunyaviridae *are known to be cleaved by SKI-1/S1P, an enzyme that normally plays a role in regulation of the lipid metabolism of the cell [6,8-10]. Fusiogenic glycoproteins of most other enveloped viruses are proteolytically cleaved C-terminally of a single arginine residue or at a multibasic recognition motif by the subtilase furin [11].

N-glycans play not only an important role in folding and intracellular transport of viral glycoproteins but also are known to modulate their antigenicity and activity [12-21]. Glycoproteins of arenaviruses vary considerably in number and position of potential N-glycosylation sites as shown by an N-glycosylation prediction program (Table 1). The Lassa virus glycoprotein GP-C contains eleven potential N-glycosylation consensus sites (N-X-S/T, in which X can be any amino acid except proline), where seven of these are located in the GP1- and four in the GP2-subunit (Table 1). Using limited N-Glycanase digestion, Wright et al. [22] demonstrated for LCMV GP that five potential glycosylation sites on GP-1 were utilized and two of the three sites on GP-2. However, for Lassa virus GP it has not yet been shown which sites are actually used and how N-glycosylation affects the characteristics of the glycoprotein in respect to cleavage and transport along the exocytotic pathway. To address this question, we investigated in the present report each potential N-glycosylation site of the Lassa virus glycoprotein concerning N-glycan maturation, cleavage of GP-C and glycoprotein transport to the cell surface. We provide evidence that each of the 11 N-glycosylation sites is used for N-glycan attachment. However, seven N-glycan mutants failed in their ability to get proteolytically processed, while 4 N-glycosylation sites are individually dispensable for cleavage of GP-C. Furthermore, we demonstrate that both uncleaved precursor and cleavage-defective N-glycan mutants are transported to the cell surface and remain mannose-rich.

**Table 1:** *Comparison of potential N-glycosylation sites between different Arenavirus glycoproteins*. Numbers indicate amino acid (aa) position of serine/threonine of potential N-glycosylation sites in Arenavirus glycoproteins. Gene bank accession numbers for viral sequences: Lassa-Josiah (J04324), LCMV-Arm (P09991), Mopeia (M33879), Tacaribe (NC_004293), White Water Arroyo (AF228063), Guanarito (AY129247), Sabia (U41071), Junin (D10072), Machupo (AY129248), Pichinde (AF081554).

	**Potential N-glycosylation sites in Arenavirus glycoproteins**																								
	**GP-1**																	**GP-2**							

**Virus**																	**N GP-1**						**N GP-2**	**N GP-C**	**aa length GP-C**

Lassa	-	-	-	81	91	101	111	-	-	121	-	-	169	-	-	226	**7**	-	367	375	392	397	**4**	**11**	**491**
LCMV	-	-	-	87	97	-	116	-	-	126	-	-	173	-	-	234	**6**	-	373	-	398	403	**3**	**9**	**498**
Mopeia	-	-	-	80	90	100	110	-	-	120	-	-	168	-	-	224	**7**	-	365	373	390	395	**4**	**11**	**489**

Machupo	-	-	-	85	97	-	-	-	-	139	168	-	180	-	-	-	**5**	-	370	378	395	400	**4**	**9**	**496**
Tacaribe	-	-	-	85	97	-	-	-	-	-	166	-	178	-	-	-	**4**	-	357	365	382	387	**4**	**8**	**483**
Sabia	-	71	-	-	90	101	-	-	127	-	-	173	180	-	-	224	**7**	-	362	370	387	392	**4**	**11**	**488**
Guanarito	-	-	-	-	90	-	-	-	127	-	-	-	176	204	-	216	**5**	316	353	361	378	383	**4**	**9**	**479**
Junin	-	-	-	-	97	107	-	-	-	-	-	-	180	-	-	-	**3**	-	359	367	384	389	**4**	**7**	**495**
Whitewater	-	-	75	-	90	-	-	-	128	-	167	-	178	-	-	217	**6**	317	354	362	379	384	**5**	**11**	**480**
Pichinde	69	76	-	91	102	113	118	123 134		-	-	-	183	-	219	243	**11**	-	381	389	406	411	**4**	**15**	**508**

## Materials and methods

### Cell culture

Vero cells were cultured in Dulbecco's Modified Eagle's Medium (DMEM, GIBCO) supplemented with 10% fetal calf serum (FCS), 100 units/ml penicillin, and 0.1 mg/ml streptomycin.

### Antibodies

Oligopeptide comprising the amino acids 477–491 of preGP-C was chemically synthesized and covalently linked to keyhole limpet hemocyanin (KLH, Pierce) as a carrier protein by the cross-linker agent N- [α-maleimidoacetoxy] succinimide ester (AMAS) and used for repeated immunization of rabbits as described before [7]. The obtained antiserum Rb-α-GP2 was tested by peptide standard ELISA [18] and used for immunoblot analyses.

### Mutation and vectorial expression of recombinant Lassa virus glycoprotein

The full length glycoprotein of Lassa virus (strain Josiah) was expressed using the beta-actin promotor-driven pCAGGS vector [6,23]. Lassa virus N-glycosylation mutants were generated by recombinant PCR using overlapping oligonucleotides, which will be made available on request. Sequences of mutants were confirmed by DNA sequencing. Vero cells were transfected with wild type and mutated recombinant DNA using Lipofectamine 2000 (Gibco/Invitrogen).

### Cell surface biotinylation assay, immunoprecipitation and treatment with glycosidases

Wild type glycoprotein of Lassa virus or N-glycosylation mutants were expressed in Vero cells. At 24 h post-transfection, cells were washed three times with cold phosphate-buffered saline (PBS) and then cell surface was incubated twice for 15 min at 4°C with 2 mg/ml sulfo-N-hydroxysuccinimidobiotin (Pierce) by adding 1 ml of the biotinylating reagent. After biotinylation, cells were washed twice with cold PBS containing 0.1 M glycine and three times with PBS. Cells were lysed in 0.5 ml RIPA (1% Triton X-100, 1% sodium deoxycholate, 0.1% SDS, 0.15 M NaCl, 10 mM EDTA, 10 mM iodacetamide, 1 mM phenylmethylsulfonyl fluoride, 50 units/ml aprotinin, and 20 mM Tris-HCl), followed by centrifugation for 30 min at 20 000 g. The supernatant was immunoprecipitated with anti-GP-C at a final dilution of 1:1000. After addition of 30 μl protein A-Sepharose CL-4B (Sigma), immunocomplexes were washed three times with RIPA. Then samples were treated with either endo-β-*N*-acetylglucosaminidase H (endo H; New England Biolabs) or N-glycosidase F (PNGase F; New England Biolabs) according to the instructions of the manufacturer, or left untreated. Samples were resuspended in reducing sample buffer for SDS-polyacrylamide gel electrophoresis (PAGE), followed by separation on a 12% polyacrylamide gel, before proteins were blotted to nitrocellulose. Biotinylated cell surface proteins were detected by incubation with a streptavidin-biotinylated horseradish peroxidase complex (Amersham Pharmacia Biotech) diluted 1:2000 in PBS. Proteins were visualized with the enhanced chemiluminescence system (Amersham Pharmacia Biotech) by exposure to an autoradiography film (Kodak BIOMAX).

## Results and discussion

### Mutation of potential N-glycosylation sites of Lassa virus preGP-C

**Figure 1:**
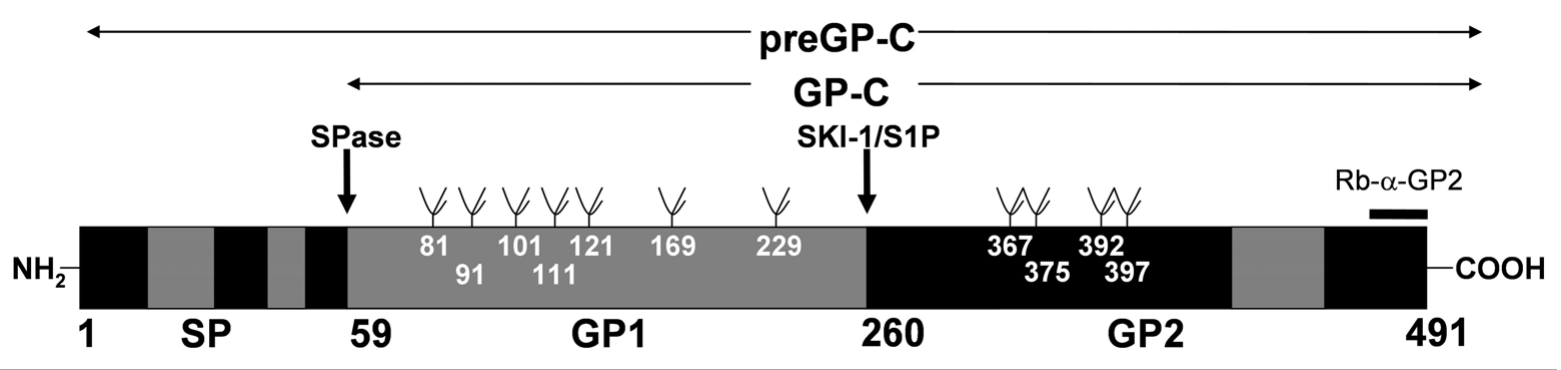
***Schematic diagram of Lassa virus glycoprotein*. **The primary translation product preGP-C (aa 1–491), the signal peptide (SP) (aa 1–58), the precursor glycoprotein GP-C (aa 59–491) and the subunits GP-1 (aa 59–259) and GP-2 (260–491) are shown. Hydrophobic regions are indicated in stripes. The signal peptidase (SPase) cleavage site between threonine residues 58 and 59 (arrow), the SKI-1/S1P cleavage site C-terminally of leucine 259 (arrow) and the rabbit antiserum binding site, α-GP2 (aa 477–491) are indicated. Eleven potential N-glycosylation sites (tree-like symbols) were identified. Amino acid positions of potential N-glycosylation sites are shown.

**Figure 2:**
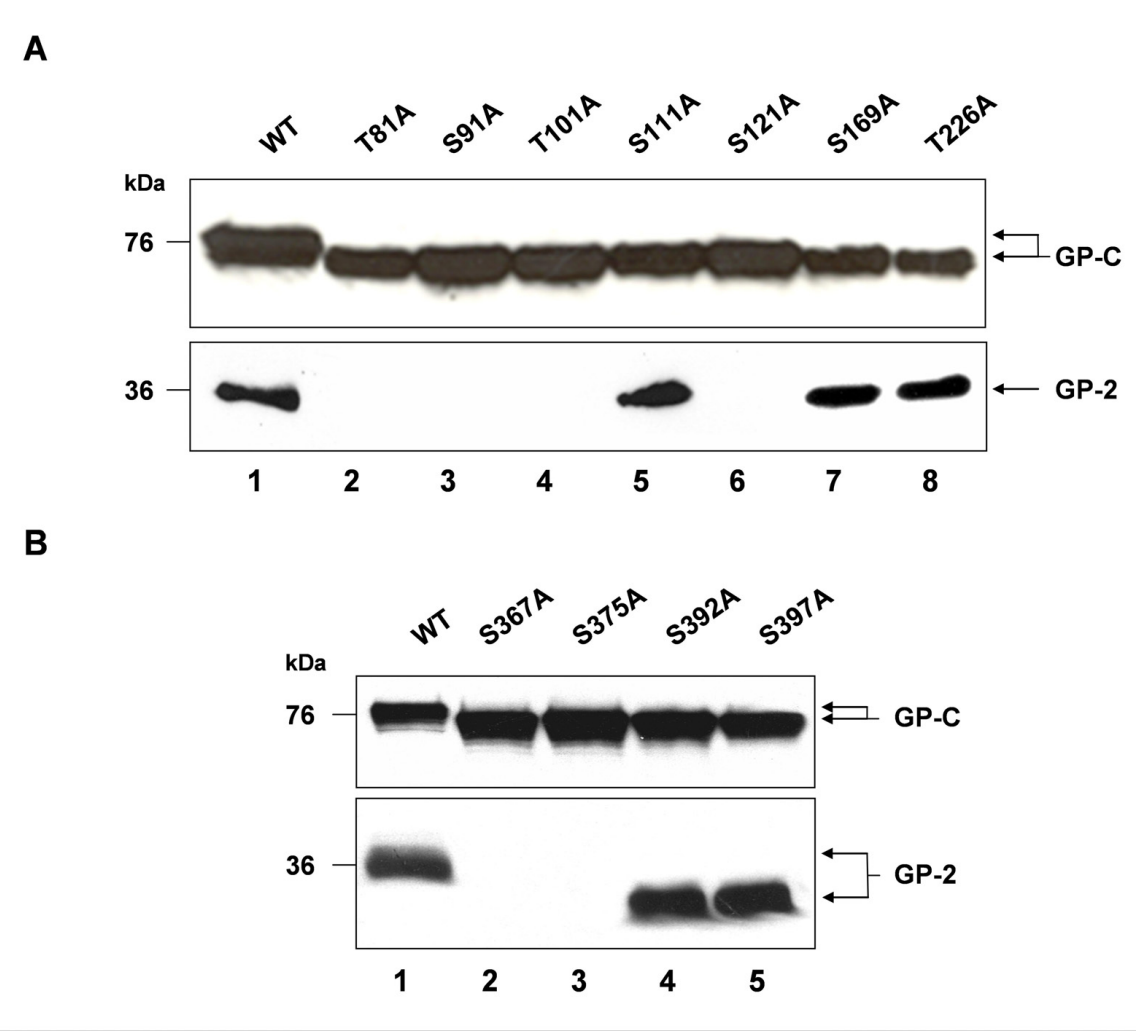
***Proteolytic processing of N-glycosylation mutants*. A **Vero cells expressing either wild-type GP-C or GP-1 N-glycosylation mutants T81A, S91A, T101A, S111A, S121A, S169A and T226A. **B **Vero cells expressing either wild-type GP-C or GP-2 N-glycosylation mutants S367A, S375A, S392A and S397A. The expressed proteins were separated by SDS-PAGE on 12% acrylamide gels and immunoblotted. Non-cleaved GP-C and its cleaved form GP-2 are detected by immunostaining using the antiserum rb-α-GP-2.

Lassa virus preGP-C contains 11 potential N-glycosylation sites, 7 in the GP-1- and 4 in the GP-2-subunit (Fig. 1). To investigate whether each N-glycosylation site is used for N-glycan attachment, the corresponding serine or threonine residues of potential N-glycosylation motifs (N-X-S/T) were individually changed to alanine. GP-1 mutants were designated T81A, S91A, T101A, S111A, S121A, S169A and T226A and GP-2 mutants S367A, S375A, S392A and S397A. All cDNA constructs were expressed transiently in Vero cells using the beta-actin promoter-driven mammalian expression vector pCAGGS. Western blotting was performed using antiserum directed against the C-terminus of GP-2. As shown in Fig. 2A (lanes 2–8, upper panel) and Fig. 2B (lanes 2–5, upper panel), all mutants showed faster migration on a SDS gel compared to wild-type GP-C indicating that each N-glycosylation site is used for N-glycan attachment. Next we determined the effect of the individual N-glycans on the maturation cleavage by analysing the presence of the cleaved subunit GP-2. Disruption of the N-glycosylation sites within the GP-1 mutants S111A, S169A and T226A has no influence on GP-C cleavage (Fig. 2A, lanes 5, 7 and 8, lower panel). In contrast, the lack of N-glycan attachment of the GP-1 mutants T81A, S91A, S101A and S121A abolished the proteolytical processing by SKI-1/S1P (Fig. 2A, lanes 2–4 and 6, lower panel). Among the four N-glycosylation sites of GP-2, only GP-C of the mutants S392A and S397A are further cleaved into GP-1 and GP-2 similar to fully glycosylated wild-type GP-C (Fig. 2B, lanes 4 and 5, lower panel), whereas no cleavage of the glycoprotein was observed within the mutants S367A and S375A (Fig. 2B, lanes 2 and 3, lower panel). Due to the high resolution of the acrylamid gel electrophoresis, the GP-2 subunit of mutants S392A and S397A revealed significantly faster electrophoretic mobility than wild type GP-2 (Fig. 2B; lanes 4–5, lower panel) showing that the respective N-glycosylation sites are used for N-glycan attachment.

### Cell surface transport of GP N-glycosylation mutants

**Figure 3:**
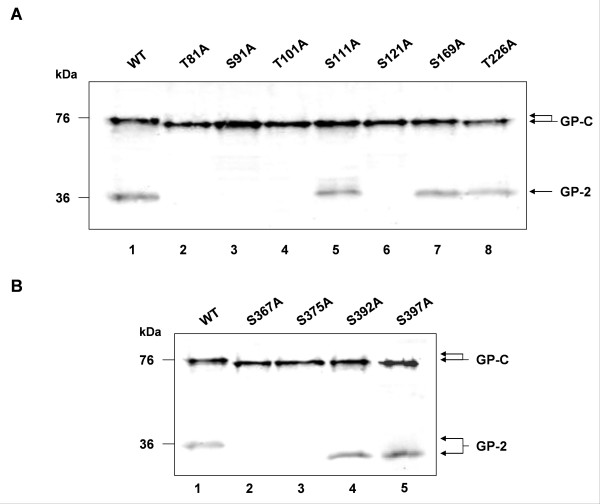
***Cell surface transport of GP-C N-glycosylation mutants*. **Vero cells were transfected with GP-1 (**A**) and GP-2 (**B**) N-glycosylation mutants of Lassa virus glycoprotein. At 24 h post-transfection, cells were surface labelled with biotin. Following cell lysis and immunoprecipitation using α-GP-2, samples were subjected to SDS-PAGE and blotted to nitrocellulose. Surface biotin-labelled proteins were visualized with streptavidin-peroxidase and chemiluminescence.

N-glycosylation has long been known to be essential for correct folding and intracellular transport of viral glycoproteins, as shown for instances for influenza hemagglutinin [19]. In order to investigate the importance of the individual N-glycosylation sites of Lassa virus GP-C for intracellular trafficking we analysed cell surface expression of the mutated glycoproteins using a biotinylation approach. Transfected Vero cells were labelled with non-membrane-permeating biotin as described in material and methods. After biotinylation, cells were lysed and GP-C and GP-C N-glycan mutants were immunoprecipitated using polyclonal antiserum directed against the C-terminus of GP-C. The precipitates were separated on an SDS gel and transferred to nitrocellulose. Surface-expressed glycoprotein protein was then visualised with a streptavidin-peroxidase complex. Fig. 3A (lanes 2–8) and B (lanes 2–5) demonstrate that all GP-1 and GP-2 N-glycosylation mutants are transported to the cell surface in a similar manner compared to wild-type GP indicating that a loss of a single N-glycosylation attachment site and proteolytic cleavage are not necessary for cell surface transport of GP. For LCMV it was shown that maturation cleavage deficient mutants are efficiently transported to the cell surface [10]. Both cleavage and cell surface expression of LCMV GP-C are impaired in parallel either when no N-glycosylation occurs or a proline residue is present at position 110 [22,24]. Since many cleavage deficient mutants have been shown to exhibit regular cell surface transport, the two reported cases in which cell surface expression was abolished might be due to misfolding in the endoplasmic reticulum with subsequent degradation [22,24].

### Transport of endo H-sensitive GP-C to the cell surface

**Figure 4:**
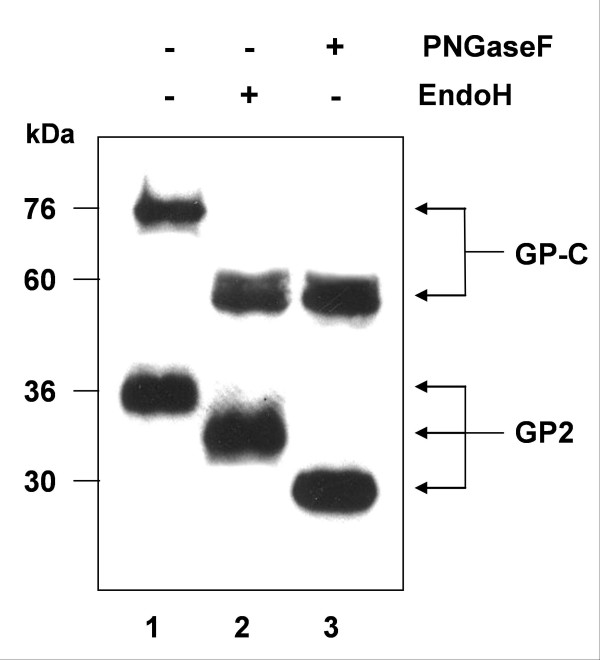
***Glycosidase sensitivity of cell surface transported Lassa virus glycoprotein*. **At 48 h post-transfection, Vero cells expressing wild-type Lassa virus glycoprotein were surface biotinylated and immunoprecipitated as described under Fig. 3. Precipitated samples were either left untreated (lane 1), digested with Endo H (lane 2) or PNGase F (lane 3). The samples were separated by SDS-PAGE with subsequent immunoblotting. Biotinylated proteins were detected using streptavidin coupled to horse radish peroxidase and chemiluminescence.

LCMV and Lassa virus GP-C are cleaved by the same protease, SKI-1/S1P, although proteolytic processing of the GPs occurs in different subcellular compartments. Lassa virus GP-C is cleaved early along the secretory pathway in the ER or an early Golgi stack, whereas LCMV GP-C is cleaved in the Golgi or post-Golgi compartment [6,10,22]. Furthermore, for LCMV GP-C it has been shown that transition of mannose-rich carbohydrates to complex carbohydrates occurred before maturation cleavage [22]. In order to analyse the glycosylation status of Lassa virus glycoproteins on the outer cell membrane, cell surface biotinylation was combined with a subsequent detachment of mannose-rich N-glycans by digestion with endo H or removal of complex N-glycans using PNGase F (Fig. 4). The GP-2 subunit is detectable at the cell surface and displays partial endo H-resistance confirming a modification of its carbohydrate decoration during glycoprotein transport to the cell surface (lane 2). Interestingly, the glycoprotein precursor GP-C which is transported to the cell surface remains completely endo H-sensitive suggesting that cleavage might be a prerequisite for further complex glycosylation. In addition, uncleaved endo H-sensitive Lassa virus GP-C is also transported to the cell surface of SKI-1/S1P deficient cells (data not shown).

The finding that only an endo H-sensitive form of Lassa virus GP-C was found at the cell surface suggests that partial endo H-resistance of the Lassa virus glycoprotein is only acquired when the glycoprotein is cleaved into its subunits. This observation is in contrast to findings for the LCMV glycoprotein where an endo H-resistant form of GP-C was described [22]. However, since cleavage of Lassa virus GP-C by SKI-1/S1P occurs earlier in the exocytotic pathway than LCMV GP-C [6,10,22], cleavage of Lassa virus GP-C occurs prior to trimming of the mannose-rich N-glycans to complex sugar types.

It can not be ruled out that the use of glycosylation sites and the N-glycan trimming differs between recombinant expressed glycoproteins and GP in LASV-infected cells. It was shown recently that recombinant expressed M protein of SARS-CoV gained complex-type N-glycosylation whereas in SARS-CoV infected cells N-glycosylation of M remained endo H-sensitive [25]. However, in general a good correlation between results obtained after solitary expression and studies using the homologues viral system have been observed. To address this kind of issues, the development of a "reverse genetic system" for LASV will be necessary that would allow analysing the role of glycosylation of GP in the correct viral context.

## Conclusion

Taken together, our study suggest that individual N-linked oligosaccharides of the Lassa virus glycoprotein differ greatly in terms of their importance for correct protein folding which seems to be important for activation cleavage by SKI-1/S1P. The differences between the precursor glycoprotein GP-C and the cleaved subunits GP-1 and GP-2, not only with respect to N-glycosylation, remain an enigma since only the cleaved glycoprotein subunits are incorporated into virus particles [6].

## Abbreviations

aa; amino acid; DMEM, Dulbecco's Modified Eagle's Medium; GP, glycoprotein, PCR, polymerase chain reaction; Endo H, endoglycosidase H; PNGase F, peptide-N-glycosidase F; RIPA, radio immunoprecipitation assay; SKI-1/S1P, subtilisin kexin isoenzyme-1/site 1 protease; SDS-PAGE, sodium dodecyl sulfate-polyacrylamide gel electrophoresis; Tris, tris-(hydroxymethyl)-aminomethan.

## Competing interests

The author(s) declare that they have no competing interests.

## Authors' contributions

RE and TS carried out experiments, participated in the analysis of the results and drafted the manuscript. OL helped to draft the manuscript and revised it critically. WG designed the study, participated in the analysis of the results and helped to draft the manuscript. All authors read and approved the final manuscript.
